# DeepScreening: a deep learning-based screening web server for accelerating drug discovery

**DOI:** 10.1093/database/baz104

**Published:** 2019-10-11

**Authors:** Zhihong Liu, Jiewen Du, Jiansong Fang, Yulong Yin, Guohuan Xu, Liwei Xie

**Affiliations:** 1 State Key Laboratory of Applied Microbiology Southern China, Guangdong Provincial Key Laboratory of Microbial Culture Collection and Application, Guangdong Open Laboratory of Applied Microbiology, Guangdong Institute of Microbiology, Guangdong Academy of Sciences, 100 Xianlie Middle Road, Guangzhou 510070, China; 2 Division of Algorithm, Beijing Jingpai Technology Co., Ltd. 1500-1, Hailong Building Z-Park, Beijing 100090, China; 3 Genomic Medicine Institute, Lerner Research Institute, Cleveland Clinic, 9620 Carnegie Ave n building, Cleveland, OH 44106, USA; 4 Zhujiang Hospital, Southern Medical University, 253 Industrial Avenue, Guangzhou 510282, China

## Abstract

Deep learning contributes significantly to researches in biological sciences and drug discovery. Previous studies suggested that deep learning techniques have shown superior performance to other machine learning algorithms in virtual screening, which is a critical step to accelerate the drug discovery. However, the application of deep learning techniques in drug discovery and chemical biology are hindered due to the data availability, data further processing and lacking of the user-friendly deep learning tools and interface. Therefore, we developed a user-friendly web server with integration of the state of art deep learning algorithm, which utilizes either the public or user-provided dataset to help biologists or chemists perform virtual screening either the chemical probes or drugs for a specific target of interest. With DeepScreening, user could conveniently construct a deep learning model and generate the target-focused de novo libraries. The constructed classification and regression models could be subsequently used for virtual screening against the generated de novo libraries, or diverse chemical libraries in stock. From deep models training to virtual screening, and target focused de novo library generation, all those tasks could be finished with DeepScreening. We believe this deep learning-based web server will benefit to both biologists and chemists for probes or drugs discovery.

## Introduction

Deep learning is one of the most popular machine learning methods, which are composed of multiple processing layers and artificial neurons to simulate human central neural system to learn patterns of the data with multiple levels of abstraction (1). Deep learning has achieved remarkable success in a wild range of areas, such as computer vision, computer games, natural language processing and self-driving cars. Healthcare, medical diagnosis and drug discovery also benefit immensely from deep learning technologies due to the rapid increase of biomedical data (2,3). For medical diagnosis, deep learning models have achieved physician-level and exhibited a promising application outlook (4–7). In drug discovery, various machine learning approaches have been applied to speed up the compounds identification with desired pharmacodynamics and pharmacokinetic properties, which are time-consuming tasks for bench workers (8–10). Various machine learning based virtual screening webserver such as OCHEM (11), ChemSAR (12), and LBVS (13), are developed to accelerate the drug discovery. Meanwhile, machine learning methods are also widely applied in the target prediction, which is another critical issue in drug discovery (14–16). Machine learning based target prediction shows great potential in drug repositioning and mechanism of action exploration. A plenty of studies suggested that deep learning techniques have shown superior performance to other machine learning algorithms in target prediction (17), ADMET properties predictions (18–20), virtual screening (21, 22) and chemical syntheses planning (23). Those could be concluded from the benchmark testing results (24), but also the data science competitions hosted by pharmaceutical companies (25). Furthermore, another impactful application of deep learning is the *de novo* molecule design, which utilize sequences data to generate molecules with desired properties (26–30). However, the application of deep learning techniques in drug discovery and chemical biology are prohibited due to the data availability, data processing, and lacking of the user-friendly deep learning tools. Therefore, we developed a user-friendly web server with integration of the state of art deep learning algorithm, which utilizes either the public or user-provided dataset to help biologists or chemists perform virtual screening either the chemical probes or drugs for a specific target of interest.

**Figure 1:**
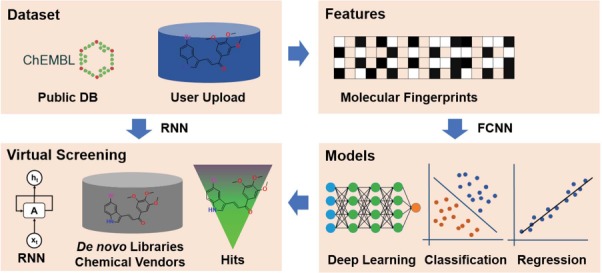
The framework of the DeepScreening. RNN: recurrent neural network. FCNN: fully connected neuron network.

## Methods

### Framework of DeepScreening

The framework of deep learning-based virtual screening server DeepScreening is simply illustrated in Figure 1. (i) Dataset Preparation: select target of interest, or upload a private dataset for deep neural network (DNN) training. (ii) Features: select features for molecular vectorization. (iii) Parameters: select model parameters for training classification or regression models. (iv) Virtual Screening: Virtual screening against chemical library or de novo library. DeepScreening is a highly automatically screening server, which integrate various technologies and tools for data processing, model construction and screening.

**Figure 2:**
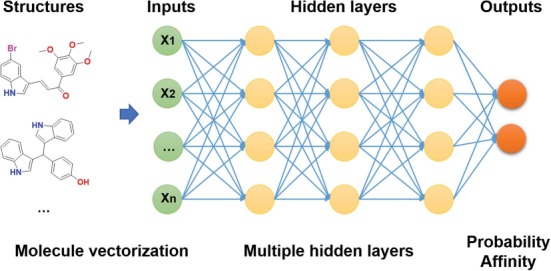
Architecture of DNNs in DeepScreening.

### Data processing

The large bioactivity database ChEMBL 24 (31) was used to extract the targets and their ligands information. First, non-molecular target types are excluded, such as ‘UNKNOWN’, ‘METAL’, ‘SUBCELLULAR’, ‘LIPID’, ‘CELL-LINE’, ‘ORGANISM’, ‘TISSUE’, ‘PHENOTYPE’, ‘ADMET’, ‘NO TARGET’, ‘UNCHECKED’. For each target, the assay confidence scores less than 8 were removed. For classification, actives were defined by IC50/EC50/Ki/Kd/Potency <10 μm (Standard relation with ‘=’, and the standard unit with ‘nM’), the remaining compounds were defined as inactives. For compounds with multiple binding affinity data, the most potent with minimal value was chosen and duplicates were removed. Finally, targets with more than 50 actives and 50 inactives in classification dataset, resulting in 1251 targets. For regression dataset, targets with more than 50 affinity data were preserved and resulting in 568 055 IC50, 70 017 EC50, 283 591 Ki and 17 078 Kd measurements across 1814 targets.

### Molecular vectorization

Molecules are required to be vectorized before the training of deep learning model. Fingerprints are popular methods for molecular representation. Here, 12 types of fingerprints employed in PaDEL (32) for molecular vectorization. The length of the fingerprints range from 79 (EState fingerprint) to 4860 (Klekota-Roth fingerprint). The details of the fingerprints were summarized in Supplementary Table S1.

### DNN training

The deep learning classification and regression models were implemented in PyTorch. Models were trained on 80% of the random split set and then validated on the remaining 20% of the dataset. The hyperparameters of the deep neuron network, such as learning rate, hidden layers, number of neurons, activation functions, dropout and batch normalizations could be varied for optimizing the model performance. The architecture of DNN in DeepScreening could be seen in Figure 2. For regression training, the binding affinity values were converted using negative log of the activity value in ChEMBL, suggesting that higher values indicate greater potency. Taking IC_50_ as example, pIC50 is the negative log of the IC50 value when converted to molar (M). When the unit of IC_50_ is nanomolar (nM), the conversion change to equation (1). For classification model, the output of DNN is the probability of a compound to be active. The hyper-parameters available in DeepScreening were listed in Supplementary Table S2.

(1) \begin{equation*} \mathrm{p}{\mathrm{IC}}_{50}=-{\log}_{10}{\mathrm{IC}}_{50}\left(\mathrm{M}\right)=9-{\log}_{10}{\mathrm{IC}}_{50}\left(\mathrm{nM}\right) \end{equation*}

### Model evaluation

In order to evaluate the model performance, various metrics have been used. For classification model, the accuracy, recall, precision, F1, Matthews correlation coefficient (MCC) and area under ROC curve (AUC) are calculated. For regression model, R squared (R^2^), mean squared error (MSE), root MSE (RMSE) and mean absolute error (MAE) were calculated. All those metrics were calculated with Sklearn. Here the best epoch during the training was defined using maximal AUC value for classification model and minimal MSE value for regression model. The best epoch rather than the last epoch for each target was saved for further screening.

**Figure 3:**
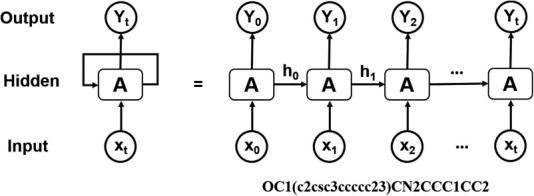
Architecture of RNN operating on the SMILES representation of molecules.

**Figure 4:**
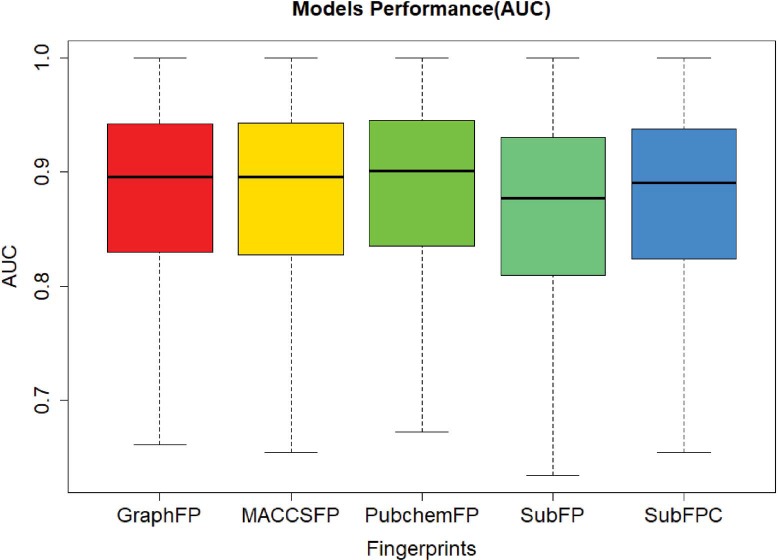
The classification model performance.

### Target-focused de novo library generation

Computational de novo drug design strategies were used to generate novel molecules with good binding affinity to the desired drug target. The application of generative models to de novo molecular design is one of the most impactful applications of DL in drug discovery. Pioneering work by Marcus *et al*. (28) demonstrated that deep recurrent neural network (RNN) operating on the SMILES representation of molecules is a promising method for molecular de novo design (Figure 3). We used the reported parameters (28), in which the RNN structure includes an embedding layer, three layers with 512 gated recurrent units for each layer, and an output linear layer. This model was trained on canonical SMILES of 1.5 million structures from ChEMBL with a batch size of 128, and utilizing the Adam optimizer. Molecules were restrained to containing between 10 and 50 heavy atoms. This model was used for randomly de novo sampling in DeepScreening. For target focused de novo sampling, the prior model was transfer trained on the new dataset with batch size of 32, learning rate 0.001, and Adam optimizer. After 10 epochs learning, target focused library was sampled from the transfer learning model. Currently, *de novo* sampling could generate chemical library with a maximal of 10 000 compounds for each job submit.

**Figure 5:**
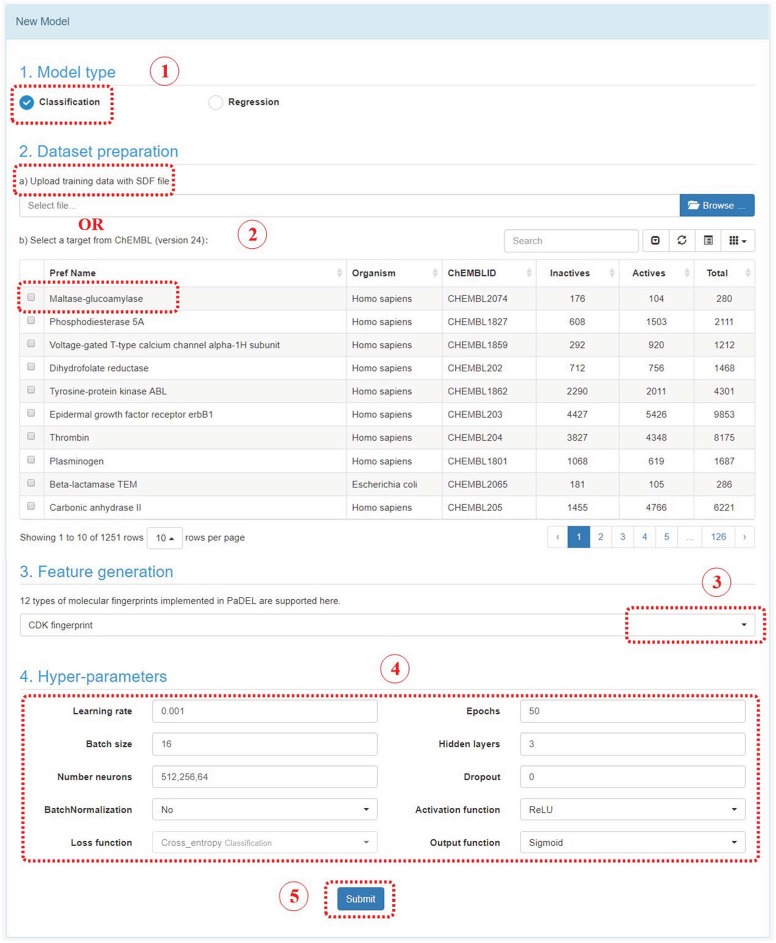
Steps to construct a deep learning model in DeepScreening.

### Web Server Implementation

DeepScreening is a publicly accessible platform, which could be accessed through a web browser using the browser/server framework. Backend was developed using Golang. The ChemDoodle web component (33) was used as a chemical structure viewer facility. The D3 library of JavaScript was used to illustrate the radical plot. Storage and management of the submitted job data were implemented by MySQL. The detailed tools for creating DeepScreening were summarized in Supplementary Table S3.

## Results

### Model performance

In order to evaluate the screening performance of the deep learning models, we selected 966 targets from the ChEMBL dataset and 5 types of fingerprints. This model contains three hidden layers with 100 neurons in each, with ReLU activation function, Adam optimizer, 0.001 learning rate and batch normalization. The best model was obtained with a maximal AUC value of validation set during 50 epochs training. The AUC values were plotted in Figure 4. The average and median of AUC were 0.86 and 0.89, respectively, which indicates a good screening performance of those constructed deep learning models.

**Figure 6:**
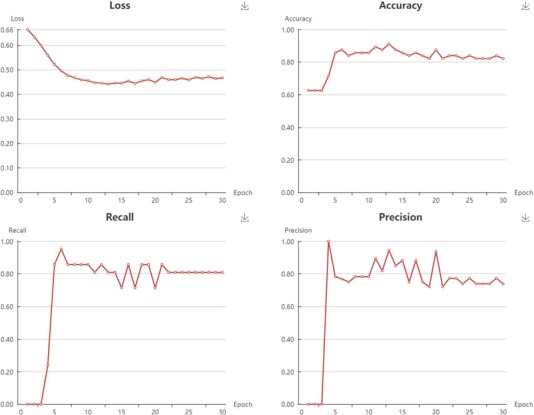
Snapshot of classification models performance.

**Figure 7:**
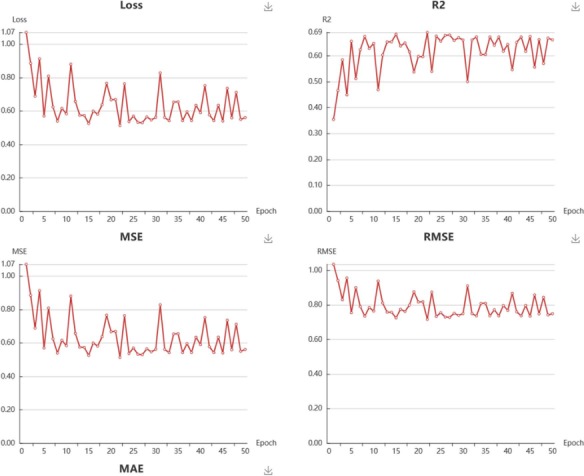
Snapshot of regressions models performance.

### Web interface

DeepScreening has three major function modules: (i) Model Module: Model submission and browsing model results; (ii) Screening Module: Virtual screening against chemical vendors or de novo libraries generated in de novo module using trained models in model module; (iii) De novo Module: random sampling or target-focused library generation.

#### Model module

In Model module, there are two sections: New Model and My Model. In New Model section, user could construct a deep learning model by following steps below (Figure 5):

Step 1: Select a model type. Either classification or regression model.

Step 2: Upload an SDF file with bioactivity information or select a target in ChEMBL. The uploaded SDF file should contain a field named ‘Activity’. For classification model, the label should be provided in this field, such as 0 stands for inactive, or 1 stands for active. For regression model, the bioactivity data should be provided with a numeric value, which stands for potency in nanomolar unit. User should convert other unit to the standard nanomolar unit before the SDF files uploaded.

Step 3: Feature generation. At present stage, 12 types of fingerprints from PaDEL are provided. The bits and descriptions of those fingerprints are listed below.

Step 4: Select hyperparameters. User could select the parameters to construct the deep neuron network. User could change the parameters and evaluate the performance of models, and find the optimum model.

Step 5: Submit. Click the submit button, which will bring you to ‘my model’ section.

In My Model section, user could browse all the models in an interactive table with searching function. In the operation filed, user could update job status. When the job is finished with a status sign of ‘Success’, user could browse the model details, screen by using current model, and download the model to local computer (Supplementary Figure S4). Click browse button, user could browse the model information and the model performance metrics. For classification model, the test loss, accuracy, recall, precision, F1 and MCC are provided (Figure 6). For regression model, the R^2^, MSE, RMSE and MAE are provided (Figure 7).

#### Screening module

In Screening module, there are two sections, New Screening and My Screening. In New Screening section, user could submit a screening through following steps (Supplementary Figure S5):

Step 1: Select a constructed model from model list or click the screening button from model list.

Step 2: Upload a chemical library with SDF file format, or select a chemical library from vendors or de novo libraries. DeepScreening has prepared a in stock diverse library covering synthetic compounds, natural products, drugs, covalent agents, PPI and allosteric modulators collected from various chemical vendors. The fingerprints of those libraries have been precalculated and are ready to screening.

Step 3: Click the submit button and turn to the My screening page to check the job status.

**Figure 8:**
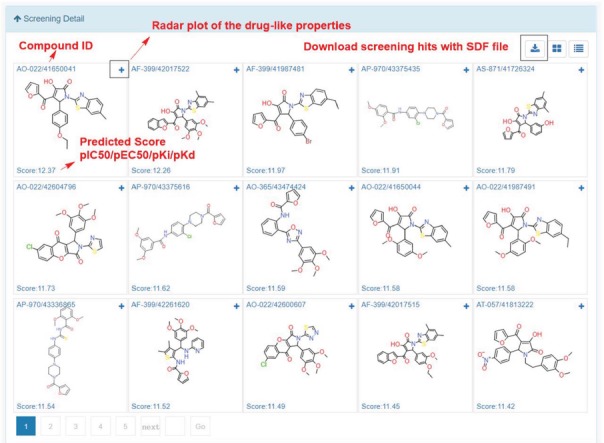
Snapshot of screening details.

**Figure 9:**
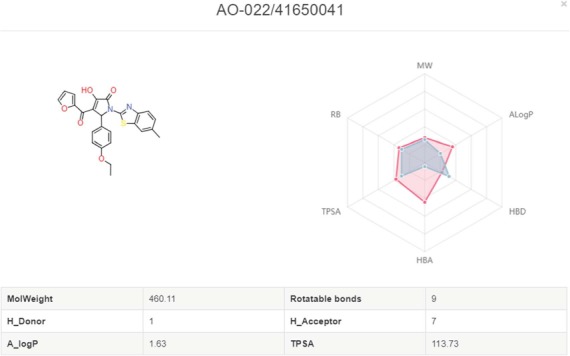
Radar plot of molecules with the drug-like properties. Red present the desired drug-like properties (MW: 500 g/mol, ALogP: 5, HBA: 10, HBD: 5, TPSA: 140, RB: 10) according to the Lipinski’s rule of 5.

In My Screening section, user could browse the screening jobs in an interactive table. When job is finished, user could click the browse button to assess the screening results, and click the download button to download the prediction results in a csv file (Figure 8). Click the screening detail button in my screening page, and turn to the ‘Screening Detail’ section as shown below. There are grid view and table view for screening hits. The Compound ID is located at up-left, and the predicted score is located at down-left. For classification models, the scores are the probabilities (0~1). For regression models, the scores are the pIC_50_, pEC_50_, pK_i_, pK_d_ (Figure 8). Greater score indicates a better affinity. Click the plus button to check the radar plot of the drug-like properties (Figure 9). Click the up-right download button to download the SDF files with a maximal of 500 compounds.

#### *De novo* library generation module

In de novo module, user could generate a randomly de novo library and target-focused library through transfer learning on a new targeted dataset, which could be selected from ChEMBL or provided by users (Supplementary Figure S6). After submitting a de novo task, user could browse all the de novo libraries in an interactive table with searching function. In the operation filed, user could update job status. When the job is finished with a status sign of ‘Success’, user could browse the library details, and download the library to local computer (Supplementary Figure S7).

### Case studies

#### Case study 1: Phosphodiesterase 4 inhibitors prediction

**Figure 10:**
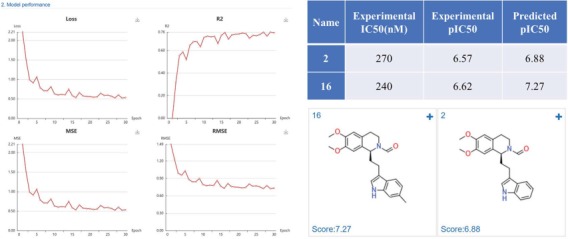
PDE4D inhibitors regression model performance and the prediction results of PDE4D inhibitors.

Phosphodiesterase 4 (PDE4) is drug target of inflammatory diseases, such as chronic obstructive pulmonary disease, psoriasis, and atopic dermatitis. Recently, Zhang *et al.* (34) reported the discovery and optimization of Tetrahydro-isoquinolines as novel PDE4D inhibitors. The virtual screening hit compound 2 (IC_50_ = 0.27 μM) and optimized lead compound 16 (IC_50_ = 0.24 μM) were selected to test the application of DeepScreening. We first constructed the regression model for PDE4D inhibitors using the IC50 data (657 compounds) in ChEMBL and using CDK fingerprint with default neuron network parameters. The model shows a good performance with a R^2^ of 0.76. Then the structure of compound 2 and 16 were submitted to DeepScreening and predicted the IC50 using the constructed model. The predicted results could be seen in Figure 10. The predicted pIC_50_ values were close to the experimental value, which suggest a high prediction accuracy of the regression model for PDE4D inhibitors.

#### Case study 2: Methionine aminopeptidase-2 inhibitors

**Figure 11:**
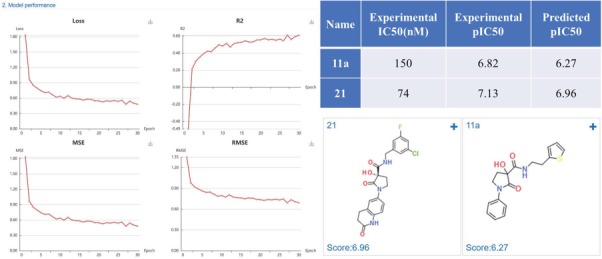
MetAP-2 inhibitors regression model performance and the prediction results of MetAP-2 inhibitors.

Methionine aminopeptidase-2 (MetAP-2) is an attractive target for anticancer therapy. Timo *et al.* (35) reported a lead compound 21 (IC_50_ = 74 nM), which was structural optimized from a high-throughput screening hit 11a (IC_50_ = 150 nM). We constructed regression model based on the MetAP-2 inhibitors IC_50_ data (553 compounds) using a default CDK fingerprint with default neuron network parameters. The model shows good performance with R^2^ of 0.6. The predicted scores (pIC_50_) were close to the experimental data, indicating a high prediction accuracy of the developed MetAP-2 inhibitors deep learning model (Figure 11).

#### Case study 3: Estrogen receptor alpha actives screening

**Figure 12:**
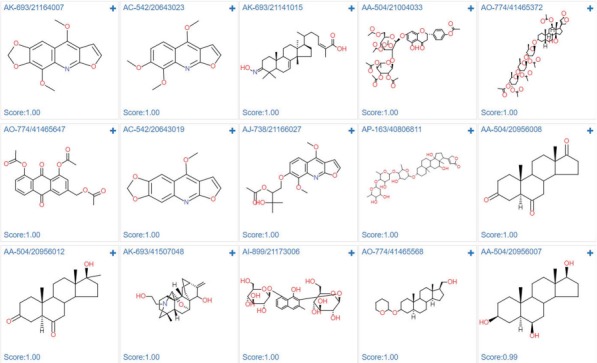
Screening results of estrogen receptor alpha model against specs natural product library.

In order to demonstrate the screening application, we select the ‘estrogen receptor alpha’ in ChEMBL with target ID of CHEMBL206, and using Pubchem fingerprints as feature method, using three hidden layers with 100 neurons in each, ReLU activation function, Adam optimizer and 0.001 learning rate. The model achieves a 0.8292 accuracy with an AUC value of 0.8506 in epoch 22, which indicates a good model. The metrics during the training epochs could be seen in Supplementary Table S8. This trained model is further applied to screening the Specs natural products library (Library ID: L00001). The screening process could be finished in several second. As shown in Figure 12, among the top ranked 15 hits, the steroids like structures (AK-693/21141015, AO-774/41465372, AP-163/40806811, AA-504/20956008, AA-504/20956012, AO-774/41465568, AA-504/20956007) and flavones (AA-504/21004033, AO-774/41465647) are identified. These results were consistent with the experimental results (36,37). Based on the prediction results, user could directly buy the chemicals from vendors using the catalog number to conduct the further bioassay (Supplementary Table S9).

## Conclusions

Although the powerful performance of deep learning technology in drug discovery is widely recognized in both academia and industry, the satisfactory user-friendly tools are still limited. In current work, we presented DeepScreening, a highly automated and efficient deep learning-based virtual screening web server, which integrates the large public bioactivity database and the state of art deep learning algorithm. With DeepScreening, user could quickly construct deep learning models, de novo generates a chemical library and use those models for virtual screening. To some extent, DeepScreening still has some limitations. Currently, DeepScreening only support bioactivity data from ChEMBL, and only fully connected neuron network are supported for virtual screening. In the future, more public dataset and more deep neuron network approaches will be added to DeepScreening to fully exploit the potential of big data and deep learning. DeepScreening aims to be the first open accessible deep learning-based virtual screening platform and more deep learning architectures will be implemented in this platform. We believe this web server would benefit the identification of bioactive compounds and facilitate the drug discovery.

## Supplementary Material

2019-07-15-SI_baz104

## Notes

**Database URL**: http://deepscreening.xielab.net
