# Supplementary material for: DeepScreening: a deep learning-based screening web server for accelerating drug discovery
Source: Database (Oxford). 2019 Oct 11;2019:baz104. doi: 10.1093/database/baz104 (PMC6790966; doi:10.1093/database/baz104)
Supplement: 2019-07-15-SI_baz104 [file 2019-07-15-si_baz104.docx]

Supplemental materials

DeepScreening: a deep learning-based screening web server for accelerating drug discovery

Zhihong Liu^1,#^, Jiewen Du^2,#^, Jiansong Fang^3^, Yulong Yin^1^, Guohuan Xu^1^, Liwei Xie*^,1,4^

^1^State Key Laboratory of Applied Microbiology Southern China, Guangdong Provincial Key Laboratory of Microbial Culture Collection and Application, Guangdong Open Laboratory of Applied Microbiology, Guangdong Institute of Microbiology, Guangdong Academy of Sciences, Guangzhou 510070, China.

^2^Beijing Jingpai Technology Co., Ltd. 1500-1, Hailong Building Z-Park, Beijing, 100090, China

^3^Genomic Medicine Institute, Lerner Research Institute, Cleveland Clinic, Cleveland, OH 44106, USA

^4^Zhujiang Hospital, Southern Medical University, Guangzhou 510282, China

*Corresponding author: E-mail: [xielw@gdim.cn](mailto:xielw@gdim.cn)

# These authors contribute equally.

Supplementary Table S1. Fingerprints available in DeepScreening

| Fingerprint | Number | Remarks | Description |
| --- | --- | --- | --- |
| **CDK fingerprint** | 1024 | CDKFP | Fingerprint of length 1024 and search depth of 8 |
| **CDK extended fingerprint** | 1024 | ExtFP | Extends the Fingerprint with additional bits describing ring features |
| **Estate fingerprint** | 79 | EStateFP | E-State fragments |
| **CDK graph only fingerprint** | 1024 | GraphFP | Specialized version of the Fingerprint which does not take bond orders into account |
| **MACCS fingerprint** | 166 | MACCSFP | MACCS keys |
| **Pubchem fingerprint** | 881 | PubchemFP | Pubchem fingerprint |
| **Substructure fingerprint** | 307 | SubFP | Presence of SMARTS Patterns for Functional Group Classification by Christian Laggner |
| **Substructure fingerprint count** | 307 | SubFPC | Count of SMARTS Patterns for Functional Group Classification by Christian Laggner |
| **Klekota-Roth fingerprint** | 4860 | KRFP | Presence of chemical substructures |
| **Klekota-Roth fingerprint count** | 4860 | KRFPC | Count of chemical substructures |
| **2D atom pairs** | 780 | AP2D | Presence of atom pairs at various topological distances |
| **2D atom pairs count** | 780 | APC2D | Count of atom pairs at various topological distances |

Supplementary Table S2. Hyper-parameters available in DeepScreening

| Hyper parameters | Values |
| --- | --- |
| Learning rate | 0.1, 0.01, 0.001 |
| Epochs | 30,50,100,200 |
| Batch size | 16, 32, 64, 128, 256 |
| Hidden layers | 1, 2, 3, 4, 5, 6, 7, 8, 9, 10 |
| Number neurons | 10, 50, 100, 200, 500,1000 |
| Activation function | ReLU, Sigmoid, Tanh |
| Dropout | 0, 10%, 20%, 50% |
| Batch normalization | Yes, No |
| Loss function | MSELoss, cross_entropy |
| Output function | self or sigmoid |

Supplementary Table S3. Tools used for creating DeepScreening

| # | Tools | Purpose | Link |
| --- | --- | --- | --- |
| 1 | ChEMBL24 | target and ligands source | www.ebi.ac.uk/chembl |
| 2 | ChemDoodle Web | structure draw and 3D display | web.chemdoodle.com |
| 3 | PaDEL | fingerprints and properties calculation | padel.nus.edu.sg/software/padeldescriptor |
| 4 | Pytorch | Deep learning package | pytorch.org |
| 5 | REINVENT | de novo design | github.com/MarcusOlivecrona/REINVENT |
| 6 | MySQL | storage database | www.mysql.com |
| 7 | Golang | web server language | golang.org |
| 8 | sklearn | model performance metrics calculation | scikit-learn.org |
| 9 | Echarts | chart visulization | echarts.baidu.com |
| 10 | bootstrap | table and web visualization | getbootstrap.com |


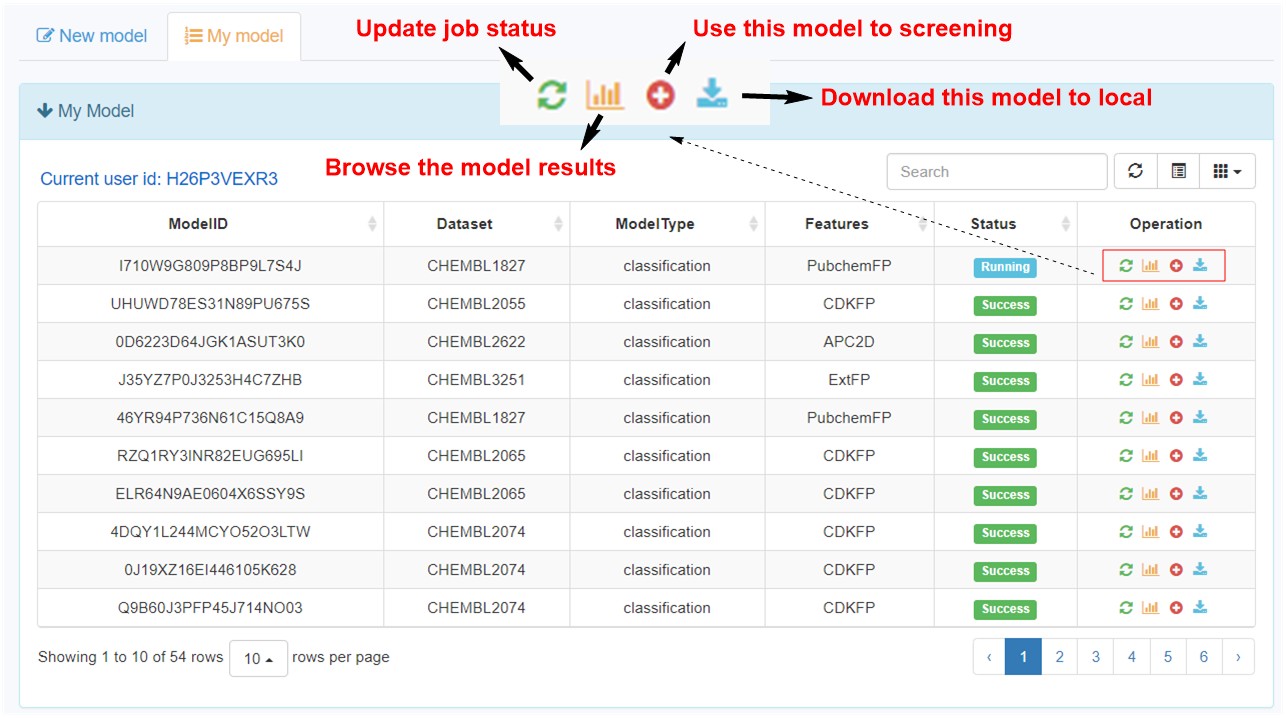


Supplementary Figure S4. Snapshot of My model section.


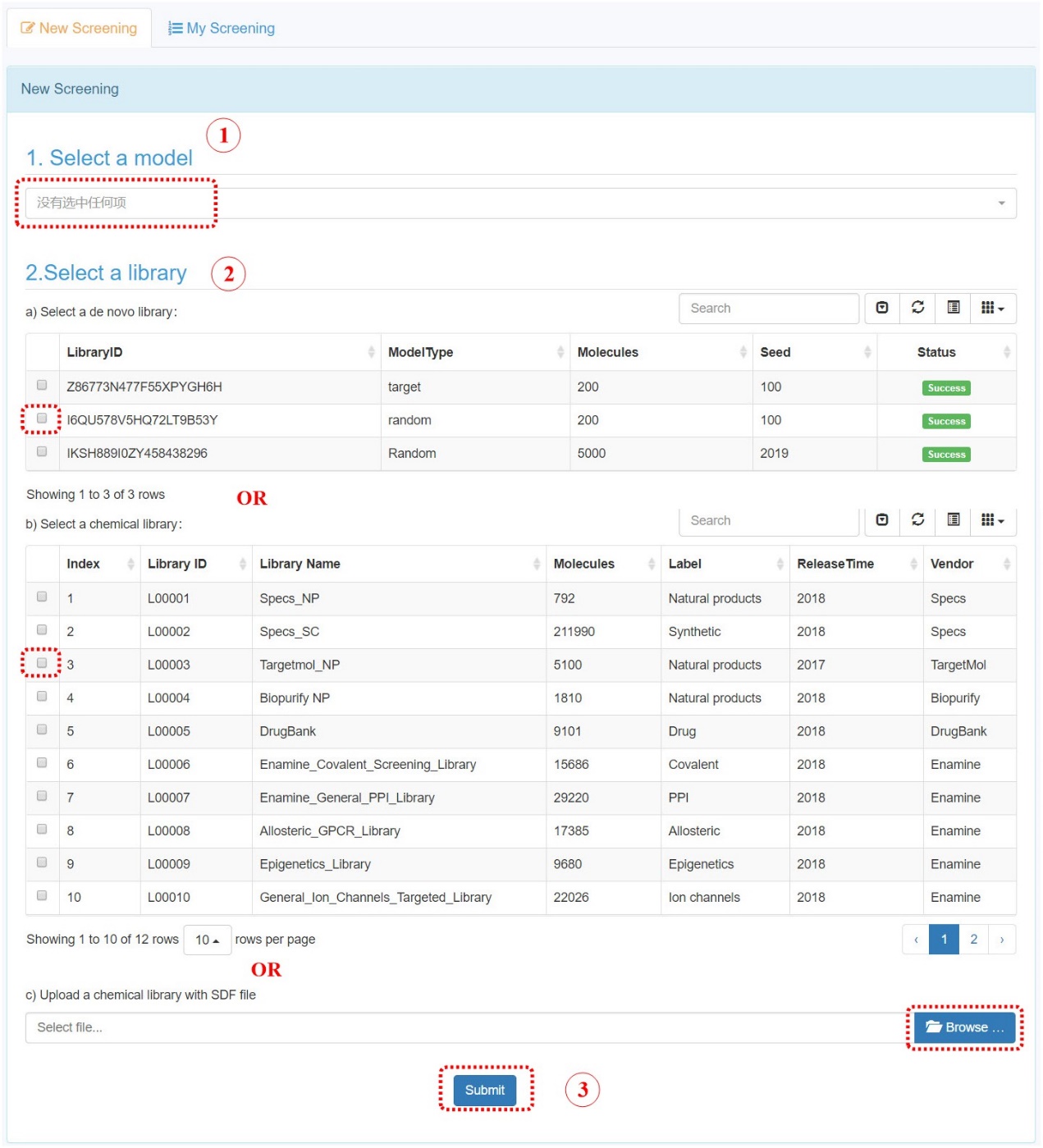


Supplementary Figure S5. Steps to submit a screening job.


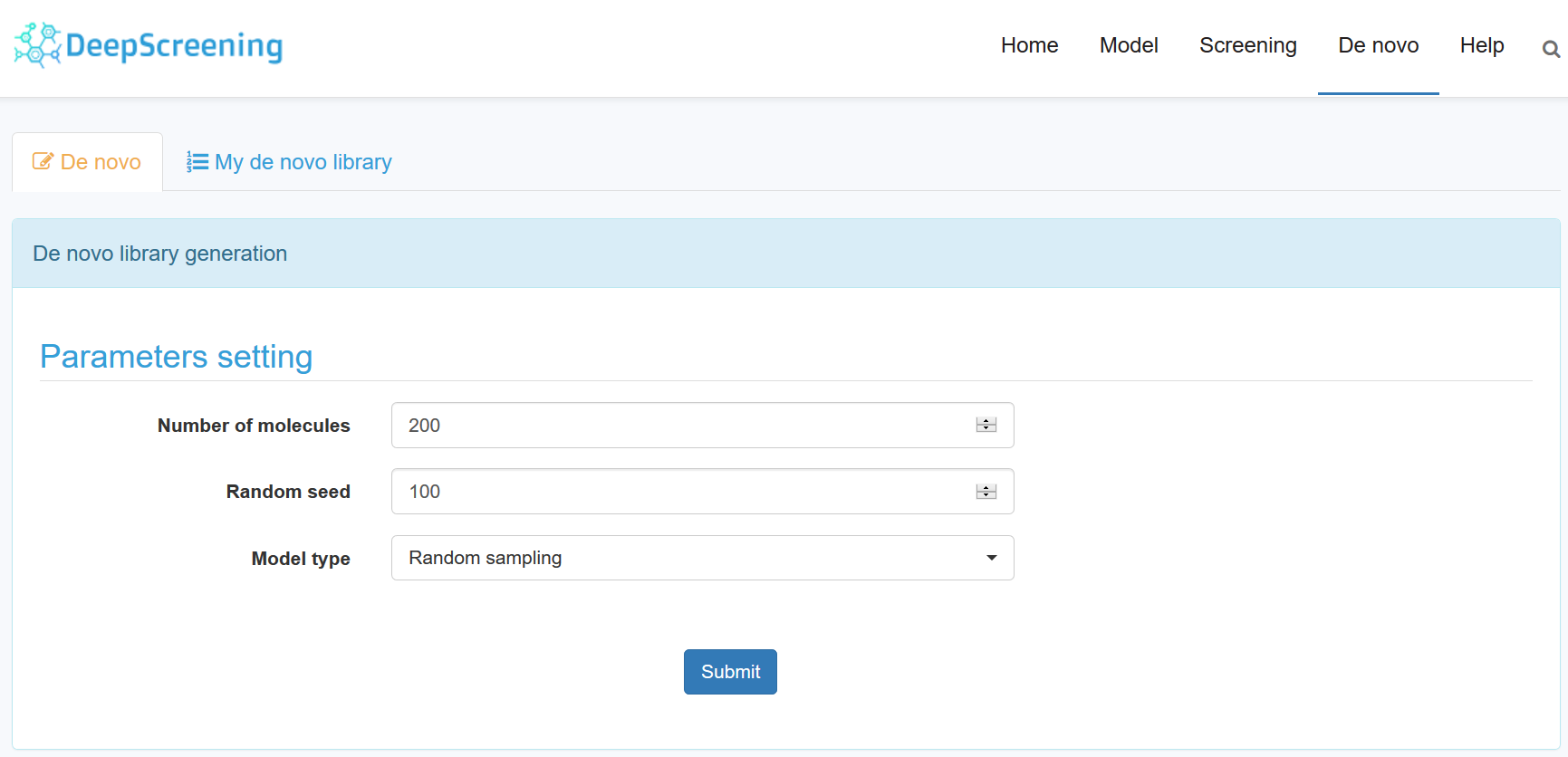


Supplementary Figure S6. Snapshot of de novo submission.


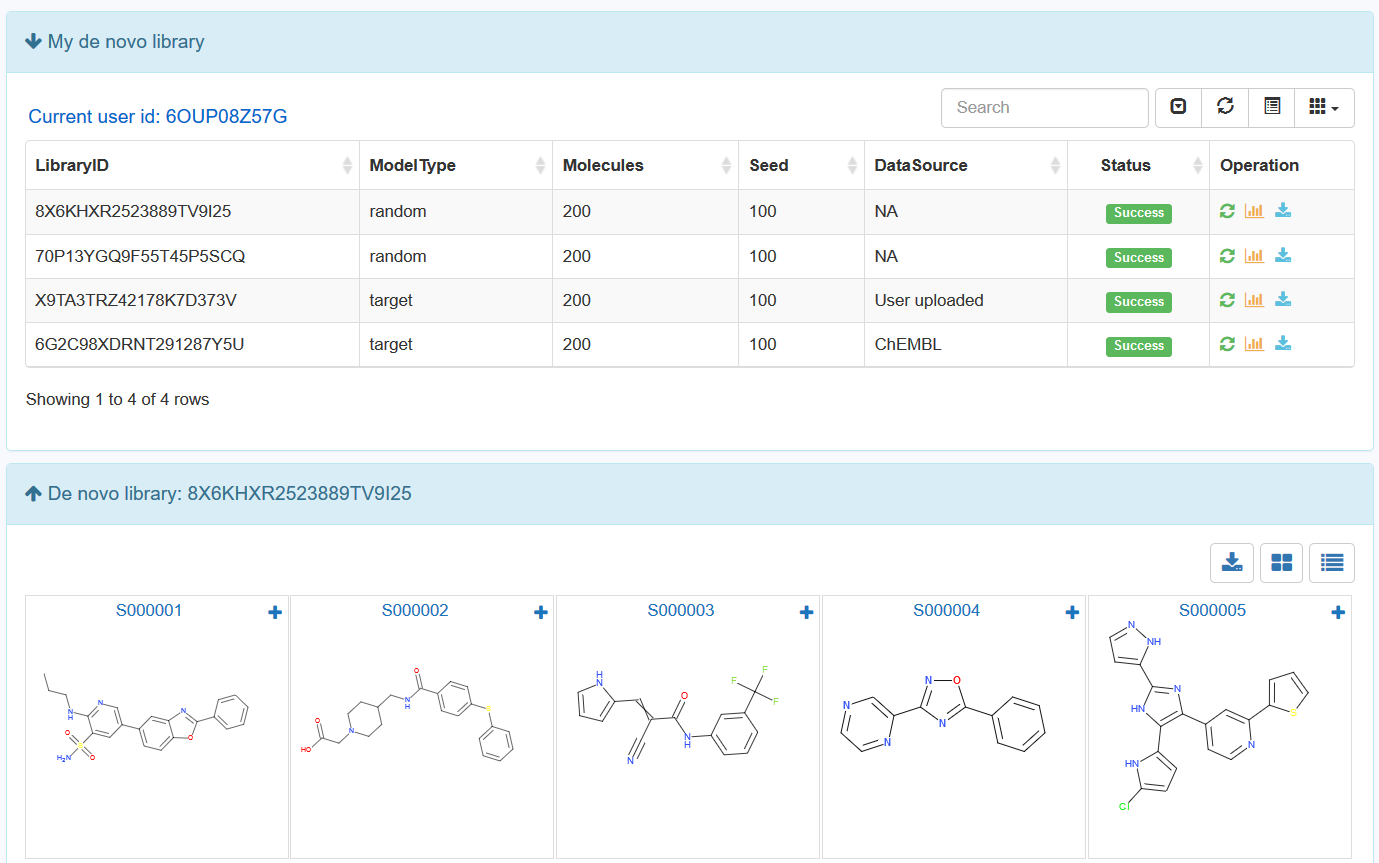


Supplementary Figure S7. Snapshot of de novo library results.

Supplementary Table S8. The model performance metrics for classification model of Estrogen receptor alpha (CHEMBL206).

| Epochs | Loss | Accuracy | Recall | Precision | F1 | MCC | AUC |
| --- | --- | --- | --- | --- | --- | --- | --- |
| 1 | 0.581668 | 0.7115 | 0.8333 | 0.6186 | 0.7101 | 0.4545 | 0.8254 |
| 2 | 0.523406 | 0.7782 | 0.6829 | 0.7682 | 0.723 | 0.5418 | 0.8376 |
| 3 | 0.521085 | 0.7861 | 0.7292 | 0.7572 | 0.7429 | 0.5601 | 0.8371 |
| 4 | 0.514967 | 0.792 | 0.6875 | 0.7941 | 0.737 | 0.5704 | 0.8349 |
| 5 | 0.525505 | 0.7772 | 0.7153 | 0.7482 | 0.7314 | 0.5416 | 0.8293 |
| 6 | 0.511973 | 0.7939 | 0.7361 | 0.7681 | 0.7518 | 0.5761 | 0.8398 |
| 7 | 0.515521 | 0.7939 | 0.7616 | 0.7546 | 0.7581 | 0.5786 | 0.8395 |
| 8 | 0.520469 | 0.7861 | 0.6134 | 0.8386 | 0.7086 | 0.5626 | 0.835 |
| 9 | 0.507035 | 0.8027 | 0.6644 | 0.8367 | 0.7406 | 0.595 | 0.8389 |
| 10 | 0.496707 | 0.8155 | 0.7245 | 0.8194 | 0.769 | 0.6196 | 0.8467 |
| 11 | 0.521767 | 0.789 | 0.7708 | 0.7416 | 0.756 | 0.5706 | 0.84 |
| 12 | 0.499951 | 0.8027 | 0.6875 | 0.8182 | 0.7472 | 0.5934 | 0.841 |
| 13 | 0.512617 | 0.7949 | 0.794 | 0.7408 | 0.7665 | 0.5851 | 0.8444 |
| 14 | 0.506904 | 0.8018 | 0.75 | 0.7751 | 0.7624 | 0.5926 | 0.8412 |
| 15 | 0.490455 | 0.8194 | 0.7222 | 0.8298 | 0.7723 | 0.628 | 0.8496 |
| 16 | 0.500751 | 0.8047 | 0.6968 | 0.8157 | 0.7516 | 0.5973 | 0.8423 |
| 17 | 0.51059 | 0.7988 | 0.7778 | 0.7551 | 0.7662 | 0.5899 | 0.8421 |
| 18 | 0.51611 | 0.787 | 0.7801 | 0.7342 | 0.7565 | 0.5684 | 0.8479 |
| 19 | 0.503383 | 0.8037 | 0.7569 | 0.7749 | 0.7658 | 0.5971 | 0.8359 |
| 20 | 0.486775 | 0.8243 | 0.7014 | 0.8584 | 0.772 | 0.64 | 0.847 |
| 21 | 0.493972 | 0.8145 | 0.7269 | 0.8156 | 0.7687 | 0.6176 | 0.8424 |
| 22 | 0.480169 | 0.8292 | 0.7454 | 0.8342 | 0.7873 | 0.6483 | 0.8506 |
| 23 | 0.500965 | 0.8077 | 0.7662 | 0.777 | 0.7716 | 0.6055 | 0.8418 |
| 24 | 0.486789 | 0.8214 | 0.706 | 0.8472 | 0.7702 | 0.6331 | 0.847 |
| 25 | 0.506797 | 0.8037 | 0.6481 | 0.8537 | 0.7368 | 0.5991 | 0.8344 |
| 26 | 0.504121 | 0.8047 | 0.6782 | 0.83 | 0.7465 | 0.5982 | 0.8397 |
| 27 | 0.497142 | 0.8096 | 0.7731 | 0.7767 | 0.7749 | 0.61 | 0.8467 |
| 28 | 0.486747 | 0.8214 | 0.7593 | 0.8079 | 0.7828 | 0.6323 | 0.8466 |
| 29 | 0.489614 | 0.8194 | 0.7083 | 0.8407 | 0.7688 | 0.6286 | 0.8455 |
| 30 | 0.497178 | 0.8096 | 0.7731 | 0.7767 | 0.7749 | 0.61 | 0.8453 |

Supplementary Table S9. The top 50 predicted score of model performance metrics for classification model of Estrogen receptor alpha (CHEMBL206).

| Name | Score |
| --- | --- |
| AK-693/21164007 | 1 |
| AC-542/20643023 | 1 |
| AK-693/21141015 | 1 |
| AA-504/21004033 | 0.9994 |
| AO-774/41465372 | 0.9993 |
| AO-774/41465647 | 0.9992 |
| AC-542/20643019 | 0.9992 |
| AJ-738/21166027 | 0.9991 |
| AP-163/40806811 | 0.9978 |
| AA-504/20956008 | 0.9969 |
| AA-504/20956012 | 0.9967 |
| AK-693/41507048 | 0.9963 |
| AI-899/21173006 | 0.9962 |
| AO-774/41465568 | 0.9957 |
| AA-504/20956007 | 0.9943 |
| AO-229/21213030 | 0.9937 |
| AO-774/41465663 | 0.9934 |
| AE-641/20167039 | 0.9931 |
| AA-504/20956019 | 0.9922 |
| AA-504/20956013 | 0.9921 |
| AC-542/20316002 | 0.9907 |
| AO-774/41465653 | 0.9874 |
| AJ-738/21061004 | 0.9827 |
| AQ-152/42730357 | 0.9825 |
| AC-542/20643020 | 0.9795 |
| AA-504/20956014 | 0.9704 |
| AJ-738/21161009 | 0.9696 |
| AO-774/41465514 | 0.9695 |
| AK-693/21087002 | 0.9689 |
| AO-774/41465578 | 0.9684 |
| AA-504/21004030 | 0.9654 |
| AA-504/07224063 | 0.9484 |
| AE-562/43462182 | 0.9424 |
| AE-508/21131014 | 0.941 |
| AJ-292/21122010 | 0.9365 |
| AK-693/40760141 | 0.9362 |
| AK-087/42718300 | 0.9176 |
| AE-842/20720002 | 0.907 |
| AA-504/21001015 | 0.8922 |
| AP-123/40765213 | 0.8644 |
| AH-034/21084004 | 0.8584 |
| AP-715/41001588 | 0.8448 |
| AO-774/41465369 | 0.8448 |
| AJ-292/21168044 | 0.837 |
| AK-693/40962738 | 0.806 |
| AE-848/32605045 | 0.8022 |
| AA-504/07225031 | 0.7783 |
| AQ-152/42730386 | 0.7734 |
| AP-163/40806740 | 0.7273 |
| AJ-160/21107003 | 0.7075 |
